# MISCORE: a new scoring function for characterizing DNA regulatory motifs in promoter sequences

**DOI:** 10.1186/1752-0509-6-S2-S4

**Published:** 2012-12-12

**Authors:** Dianhui Wang, Sarwar Tapan

**Affiliations:** 1 Department of Computer Science and Computer Engineering, La Trobe University, Melbourne, Victoria 3086, Australia

## Abstract

**Background:**

Computational approaches for finding DNA regulatory motifs in promoter sequences are useful to biologists in terms of reducing the experimental costs and speeding up the discovery process of *de novo *binding sites. It is important for rule-based or clustering-based motif searching schemes to effectively and efficiently evaluate the similarity between a *k*-mer (a *k*-length subsequence) and a motif model, without assuming the independence of nucleotides in motif models or without employing computationally expensive *Markov *chain models to estimate the background probabilities of *k*-mers. Also, it is interesting and beneficial to use a *priori *knowledge in developing advanced searching tools.

**Results:**

This paper presents a new scoring function, termed as MISCORE, for functional motif characterization and evaluation. Our MISCORE is free from: (i) any assumption on model dependency; and (ii) the use of Markov chain model for background modeling. It integrates the compositional complexity of motif instances into the function. Performance evaluations with comparison to the well-known Maximum a Posteriori (MAP) score and Information Content (IC) have shown that MISCORE has promising capabilities to separate and recognize functional DNA motifs and its instances from non-functional ones.

**Conclusions:**

MISCORE is a fast computational tool for candidate motif characterization, evaluation and selection. It enables to embed priori known motif models for computing motif-to-motif similarity, which is more advantageous than IC and MAP score. In addition to these merits mentioned above, MISCORE can automatically filter out some repetitive *k*-mers from a motif model due to the introduction of the compositional complexity in the function. Consequently, the merits of our proposed MISCORE in terms of both motif signal modeling power and computational efficiency will make it more applicable in the development of computational motif discovery tools.

## Background

Gene transcription is controlled by the essential interactions between Transcription Factor Binding Sites (TFBSs, or simply Binding Sites) and Transcription Proteins known as Transcription Factors (TFs) [1]. Understanding these interactions requires a knowledge on all binding sites associated with their TFs and *cis*-regulatory modules. Hence, discovering unknown motifs (i.e., a collection of binding sites) in co-expressed genes or finding *de novo *binding sites associated with a known TF is crucial to understand the gene regulatory mechanisms [2-4]. Experimental approaches for finding DNA motifs are laborious and expensive [5,6]. Additionally, experimental techniques such as ChIP-chip [7], ChIP-seq [8] and micro-array technology are mostly incapable of predicting specific locations of the binding sites.

It was the biological significance of the cost-effective identification of the DNA motifs that computational motif discovery has received considerable attention in the last two decades. In addition to being cost-effective and time-efficient, the nature of computational techniques offers the fastest and usually the easiest means of adopting rapidly emerging new and revised understandings on the biological process to produce more sensible motif discovery results. Despite being enormously attempted, an effective motif discovery performance by the computational approaches still remains challenging [9-11]. This is partly due to the lack of effective characterization on regulatory motifs which helps in distinguishing the functional motifs from the non-functional ones.

Due to the functional significance in gene regulation, motifs are evolutionarily conserved. Hence, motif instances appear to be rather similar to each other despite having variability in their nucleotide compositions [12]. Motif instances are rarely found in the background sequences, which is often termed as the background rareness. Also, functional motifs are often over-represented in the regulatory regions (foreground) compared to the backgrounds [13-16]. Thus, a motif's background-to-foreground appearance ratio should be smaller than the random ones. Over-representation can be similarly interpreted with the rareness characteristic. However, they are typically expressed with different statistical representations. Another useful characteristic of functional motifs is related to the compositional complexity of the nucleotides, which is termed as motif complexity [17].

Information Content (IC) [18] and Maximum a Posteriori (MAP) [19] score are two conventional motif scoring schemes that are widely adopted in evaluating and ranking candidate motifs. They are capable of characterizing the model conservation and the background rareness properties of the functional motifs. However, they suffer from the following shortcomings:

1. IC evaluates a motif by quantifying the relative entropy of the motif PFM (Positional Frequency Matrix) under assumption of model independence. This assumption on model independence is fundamentally weak as shown in [20-23].

2. MAP, on the other hand, requires a higher order Markov chain model to estimate the background probabilities [24] prior to motif evaluation. Its computational time and cost increases along with the increment of the order of the used Markov chain model. Also, MAP score can not be used to evaluate the similarity between a *k*-mer and a motif model, which is essential in computational motif discovery exercises.

3. Both IC and MAP score ignore the motif-complexity feature in the evaluation of the candidate motifs. Hence, a complexity score-based filtering [17] has to be used in candidate motif evaluation. The complexity threshold is empirically set in the filtering process that needs human intervention and careful attempts.

4. Computational motif discovery can be guided by some known motif models as useful *a priori *knowledge (*pk*). Motif evaluation in terms of ranking then becomes a motif-to-motif similarity task. Unfortunately, IC and MAP score are not able to embed the *pk *models in scoring.

Motivated by the above issues, this paper introduces a new motif scoring function, termed as MISCORE (mismatch-based matrix similarity scores), to quantify similarity between a *k*-mer and a motif PFM using a mismatch computation on the nucleotides. By evaluating each instance *k*-mer (a candidate binding site) of a motif, MISCORE can quantify the likeliness of the candidate motif to be functional by a combined characterization on the model conservation, the background rareness and the compositional complexity. Our proposed MISCORE share the following three remarkable features: (i) computational efficiency due to its simplicity; (ii) free from any assumption on model dependency; and (iii) an embedability of *a priori *knowledge in motif scoring. An extension of MISCORE, that adopts a biologically inclined pattern localization approach for an improved recognition of the functional motifs, is also reported in this paper.

Experiments on 33 benchmark DNA datasets have been carried out for evaluating the performance of MISCORE with comparison to IC and MAP score. Firstly, we examine how well these metrics can separate the functional motifs from the random ones. Secondly, we are interested in learning how well they can recognize the functional motifs from a set of putative motif models in terms of candidate ranking. Lastly, we evaluate the effectiveness of MISCORE in recognizing the functional motifs using *pk *models. The experimental results are found promisingly supportive to MISCORE.

Over-representation is a widely recognized numerical feature for characterizing functional motifs [13-15], that typically differs from the statistical quantification of the background rareness property. Due to their common objective of motif characterization, correlating them through a single framework is fundamentally meaningful and it has not been addressed sufficiently in the literature. MISCORE can be utilized as a similarity metric to perform this correlation as detailed in the latter portion of this paper.

## Methods

This section describes MISCORE and its localized version in details. For the sake of completeness, some preliminaries are given, including the notations and the *k*-mer encoding scheme used throughout this paper, followed by a preliminary introduction on the motif complexity score [17], Information Content [18] and the Maximum a Posteriori score [19].

### Preliminaries

#### Model representation

In this paper, Positional Frequency Matrix (PFM) is employed as the motif model [18]. The PFM-based motif model, denoted by *M*, is a matrix, i.e., *M *= [*f*(*b_i_*, *i*)]_4×*k*_, where *b_i _*∈ *χ *= {*A*, *C*, *G*, *T*} and *i *= 1, . . ., *k*, and each entry *f*(*b_i_*, *i*) represents the probability of nucleotide *b_i _*at position *i*. Similarly, a *k*-mer *K_s _*= *q*_1_*q*_2 _. . . *q_k _*is encoded as a binary matrix *K *= [*k*(*b_i_*, *i*)]_4×*k *_with *k*(*q_i_*, *i*) = 1 and *k*(*b_i_*, *i*) = 0 for *b_i _*≠ *q_i_*. For example, a *k*-mer *K_s _*= *AGCGTGT *can be encoded as,

$$K=encode\left(K_{s}\right)=\begin{array}{c} A\\ C\\ G\\ T \end{array}\;{\left[\begin{array}{ccccccc} 1 & 0 & 0 & 0 & 0 & 0 & 0\\ 0 & 0 & 1 & 0 & 0 & 0 & 0\\ 0 & 1 & 0 & 1 & 0 & 1 & 0\\ 0 & 0 & 0 & 0 & 1 & 0 & 1 \end{array}\right]}_{4\times k}.$$

For a given binary encoded set of *k*-mers, *S *= {*K*_1_, *K*_2_, ..., *K_P_*}, the motif PFM model *M_S _*can be computed by $M_{S}=\frac{1}{P}\sum_{i=1}^{P}K_{i}$.

#### Model complexity

Motif discovery tools often return models with low complexity, that show a repetitive occurrence of nucleotides. Hence, a motif-complexity score was proposed in [17] to filter out models with lower complexities, that is,

(1) $$c\left(M\right)={\left(\frac{1}{4}\right)}^{k}\underset{\forall b_{i}\in \chi }{\prod }{\left(\frac{k}{\sum_{i=1}^{k}f\left(b_{i},i\right)}\right)}^{\Sigma_{i=1}^{k}\;f\left(b_{i},i\right)},$$

where *k *is the length of *k*-mers and *f*(*b_i_*, *i*) is the observed frequency of the base *b_i _*at position *i *in the model *M*. Here, the complexity score lies in [(1/4)*^k^*, 1], where 1 refers to a fully complex motif PFM.

#### Maximum a posteriori (MAP) score

MAP score [19] is a powerful quantifier that evaluates the merit of a candidate motif (a set of *k*-mers, *S*) by considering its model conservation and the background rareness. The background rareness of *S *is computed using a higher order Markov chain model [24]. For each *K *∈ *S*, this model can produce an estimation of background probability, namely *p*(*K*|*B*), for a given background model *B*:

(2) $$p\left(K|B\right)=p\left(b_{1},\;b_{2},\;\ldots ,\;b_{m}\right)\overset{k}{\underset{i=m+1}{\prod }}p\left(b_{i}|b_{i-m},\;b_{i-m+1},\;\ldots ,\;b_{i-1}\right),$$

where *m *is the Markov chain order; *k *is the length of *k*-mers; *p*(*b*_1_, *b*_2_, . . ., *b_m_*) is the estimated probability of subsequence *b*_1_, *b*_2_, . . ., *b_m _*and *p*(*b_i_*|*b*_*i*-*m*_, *b*_*i-m*+1_, . . ., *b*_*i*-1_) is the conditional probability of the subsequence *b_i _*under *b*_*i*-*m*_, *b*_*i*-*m*+1_, . . ., *b*_*i*-1 _occurrence constraint. Then, for the candidate motif *S*, MAP score can be expressed as,

(3) $$MAP\left(S\right)=-\frac{\text{ln}\left(\left|S\right|\right)}{k}\left(E\left(S\right)+\frac{1}{\left|S\right|}\underset{\forall K\in S}{\sum }\text{ln}p\left(K|B\right)\right),$$

where |*S*| is the cardinality of the set *S *and *E*(*S*) is the entropy [25] of the PFM (M), expressed as,

(4) $$E\left(S\right)=-\overset{k}{\underset{i=1}{\sum }}\underset{\forall b_{i}\in \chi }{\sum }f\left(b_{i},\;i\right){\text{log}}_{2}f\left(b_{i},\;i\right).$$

A higher MAP score indicates a better likeliness of the motif *S *to be functional.

#### Information content (IC)

IC [18], measuring the average binding energy of the *k*-mers set *S*, can be given by,

(5) $$IC\left(M\right)=\overset{k}{\underset{i=1}{\sum }}\underset{\forall b_{i}\in \chi }{\sum }f\left(b_{i},\;i\right)\text{ln}\left(\frac{f\left(b_{i},i\right)}{p\left(b_{i}\right)}\right),$$

where *f*(*b_i_*, *i*) is frequency of the base *b_i _*at position *i *in the model *M*, and *p*(*b_i_*) is the pre-computed background frequency of the nucleotide base *b_i_*. A higher IC score of a candidate motif indicates a better potential of being a functional one.

### MISCORE for motif characterization

MISCORE is a new scoring function for modeling motif signals that uses a combined characterization on the model conservation, the background rareness and the compositional complexity of functional motifs. It quantifies a similarity between a *k*-mer *K *and a putative model *M *with respect to the background reference model *M_ref_*, that is,

(6) $$r\left(K,\;M\right)=\frac{d\left(K,M\right)}{d\left(K,M_{ref}\right)+c\left(K\right)},$$

where *d*(*K*, *M*) is defined as a generalized Hamming distance, expressed as,

(7) $$d\left(K,\;M\right)=1-\frac{1}{k}\overset{k}{\underset{i=1}{\sum }}\underset{\forall b_{i}\in \chi }{\sum }f\left(b_{i},\;i\right)k\left(b_{i},\;i\right),$$

where *f*(*b_i_*, *i*) and *k*(*b_i_*, *i*) are the observed frequencies of base *b_i _*at position *i *in *M *and *K*, respectively.

Motivated by the well-known Gini index to quantify impurity of data clusters, we define *c*(*K*) in Eq (6) to compute the compositional complexity of *K *as follows:

(8) $$c\left(K\right)=\frac{4}{3}\left[1-\frac{1}{k^{2}}{\underset{\forall b_{i}\in \chi }{\sum }\left(\overset{k}{\underset{i=1}{\sum }}k\left(b_{i},\;i\right)\right)}^{2}\right],$$

where the complexity is scored according to the distribution of bases (*A*, *C*, *G*, *T*) in the *K*. An equal distribution gives the maximum score of 1 and a dominant distribution, i.e., a nucleotide appears at all positions of the *K*, gives the minimum complexity of 0. In Eq (6), the score range for both *d*(*K*, *M_ref_*) and *c*(*K*) is 0[1]. The complexity measure given in Eq (6) helps in automatically eliminating the low-complex motifs from the top rank. In this way, an empirical threshold-based filtering [17] for filtering the low-complex candidate motifs can be avoided.

While no *pk *model associated with the target motifs is available, then we need to employ some searching tools to generate a model that is qualified to be an approximation *M *of the target motifs. Then, this putative model is essentially derived from the information embedded in the input sequences by the employed search algorithms. For instance, in the clustering type of motif finding algorithms [17,26,27], the putative models can be obtained by grouping *k*-mers based on a similarity metric.

Binding sites are evolutionarily constrained with limited mutations, hence a *K *can be a putative motif instance if *d*(*K*, *M*) <*d*(*K*, *M_ref_*) holds, which implies a smaller mismatch to the putative model *M *than the background reference model *M_ref_*. Note that the *M_ref _*is a PFM that can be constructed by all *k*-mers from the background sequences. For a large sized background, each column of the *M_ref _*approximates the nucleotides background frequency. Thus, the *M_ref _*can be conveniently composed of the nucleotides pre-computable background frequency in each column. Large sequence-portions that have a minimal chance of having the true binding sites can be taken as the backgrounds, e.g., random chunks of large genomic portions or a large collection of upstream regions from the relevant species. Note that a smaller *r*(*K*, *M*) score characterizes a higher similarity of that *K *to *M *in respect to its dissimilarity to *M_ref _*and a better nucleotide complexity in *K*, which implies a combined characterization on the model conservation, the background rareness and the compositional complexity.

A mathematical expectation of the MISCORE values of a set of *k*-mers can be viewed as a metric to characterize the candidate motifs. Given a set of *k*-mers *S *and its PFM model *M_S_*, a MISCORE-based Motif Score (MMS), denoted as *R*(*S*), can be evaluated by,

(9) $$R\left(S\right)=\frac{1}{\left|S\right|}\underset{\forall K\in S}{\sum }r\left(K,\;M_{S}\right),$$

where | * | is the set cardinality and *r*(*, *) is the MISCORE given in Eq (6). A smaller MMS score indicates a better potential for a candidate motif to be functional.

### Remark

Initially, MISCORE was introduced in [28] to quantify a mismatch-based similarity between a *K *and a model *M_S_*, i.e., $d\left(K,\;M_{S}\right)=k-\sum_{i=1}^{k}\sum_{\forall b_{i}\in \chi }f\left(b_{i},\;i\right)k\left(b_{i},\;i\right)$. A corresponding MMS was defined by $MMS\left(S\right)=\frac{1}{\left|S\right|}\sum_{\forall K\in S}d\left(K,\;M_{S}\right)$, and utilized as a motif scoring function to quantify the conservation property of a motif *S*. In [29], an improved version of MISCORE, termed as relative-MISCORE, was introduced to characterize a motif's conservation and the rareness properties by introducing a background reference model *M_ref _*in the MISCORE computation. Let *r*(*K*, *M_S_*) denote a relative similarity between a *K *and a model *M_S_*. Then, it can be computed by *r*(*K*, *M_S_*) = *d*(*K*, *M_S_*)/*d*(*K*, *M_ref_*) that results in a relative-MMS: $RMMS\left(S\right)=\frac{1}{\left|S\right|}\sum_{\forall K\in S}r\left(K,\;M_{S}\right)$. As a new scoring function, it was employed as a fitness function in our GAPK framework for motif discovery. In this paper, we introduce a compositional complexity term in the relative-MISCORE as shown in Eq (6), which improves our previous work by preventing *k*-mers with repetitive nucleotides from motif models. This new characterization simultaneously addresses the model conservation, background-rareness and the compositional complexity properties of the regulatory motifs, which makes the present MISCORE functionally advantageous than IC, MAP score and the previous MISCORE versions. It should be pointed out that other forms of characterization on regulatory motifs exist, provided that they can model the motif signals effectively and efficiently.

**Observation: **Experiments on real DNA datasets demonstrated that *R *scores of the functional motifs are with statistically significant *p*-values and *z*-scores, that can be computed using large collections of (i) random and (ii) conserved models, generated from the respective promoter sequences. Results obtained on 12 real DNA datasets are presented in Table 1, showing that *R *scores of the true models *M_t _*(functional motif) are mostly rare with comparison to the conserved-models *M_c_*, indicated by close to zero *p*-values. Each *M_c _*is generated by a random selection of a seed *K *from a random sequence and by collecting the most similar *Ks *to the seed, only one was picked from each sequence. It shows that, despite being conserved, *M_c _*models are rarely putative to be functional in MMS scoring as anticipated. In regard to this, *R*(*M_t_*) scores are found to be the rarest with comparison to the random models *M_r_*, which is indicated clearly by the 0.000 *p*-values and reasonably high *z*-scores. Each random model *M_r _*was composed of one randomly selected *K *from each sequence.

**Table 1:** Conservation and rareness characterization of functional motifs

*TF*	*R*(*M_t_*)	Conserved (*M_c_*) models 5000 models			Random (*M_r_*) models 5000 models		
	
		*E*{*R*(*M_c_*)} ± *std*	*p*-value	*z*-score	*E*{*R*(*M_r_*)} ± *std*	*p*-value	*z*-score
CREB	0.188	0.257 ±_0.025_	0.009	02.75	0.458 ±_0.016_	0.000	16.60
SRF	0.193	0.286 ±_0.025_	0.000	03.76	0.458 ±_0.012_	0.000	22.01
TBP	0.134	0.243 ±_0.027_	0.000	04.04	0.493 ±_0.008_	0.000	43.79
MYOD	0.104	0.195 ±_0.036_	0.004	02.54	0.467 ±_0.016_	0.000	22.22
ERE	0.214	0.331 ±_0.012_	0.000	10.15	0.439 ±_0.007_	0.000	31.87
E2F	0.203	0.309 ±_0.019_	0.000	05.65	0.444 ±_0.009_	0.000	27.54
CRP	0.307	0.380 ±_0.006_	0.000	11.48	0.422 ±_0.005_	0.000	21.45
GAL4	0.246	0.261 ±_0.016_	0.181	00.88	0.418 ±_0.008_	0.000	20.95
CREB*	0.188	0.224 ±_0.024_	0.058	01.47	0.460 ±_0.017_	0.000	15.76
SRF*	0.193	0.261 ±_0.023_	0.000	03.01	0.461 ±_0.010_	0.000	26.46
TBP*	0.134	0.186 ±_0.026_	0.010	02.03	0.491 ±_0.007_	0.000	48.37
MYOD*	0.104	0.158 ±_0.033_	0.057	01.62	0.472 ±_0.015_	0.000	24.05

Remark: the following relation *R*(*M_t_*) <*E*{*R*(*M_c_*)} <*E*{*R*(*M_r_*)} indicates the characterization of the conservation property by MISCORE, while the rareness is indicated by a smaller *p*-value and a larger *z*-score obtained by the *R*(*M_t_*) models (true models) compared to the *R*(*M_c_*) (conserved) and *R*(*M_r_*) (random) models. Here, *z*-score(*M_t_*, *M_r_*) = [*E*{*R*(*M_r_*)} - *R*(*M_t_*)]/*std*{*R*(*M_r_*)}, and *p*-value(*M_t_*, *M_r_*) = *n*/5000, where *n *is the number of the random models that can hold *R*(*M_r_*) ≤ *R*(*M_t_*). It reads similarly for the conserved models *M_c_*. *E*{*} is the mathematical expectation. Note: Datasets with asterisk are composed of promoters with 500*bp*, while the others have 200*bp *in length.

### Localized-MISCORE

Transcription proteins rarely contact a single nucleotide without interacting with the adjacent bases in the binding process. Hence, the positions with a higher binding energy given by IC (and also a lower binding energy) are usually clustered as local information blocks in the PFM model of functional motifs [30]. Position-specific similarity metrics assign an equal weight to every position in the model and ignore the variability among the local blocks appearing in the motif PFMs. Since, a motif PFM can be regarded as a descriptor of its binding preferences, the underlaying nucleotide blocks are believed to carry useful information that constitutes the overall characterization of the motif. Based on this understanding, we aim to decompose a motif PFM into a set of local blocks and assign a weight to each block according to its potential of being functional.

MISCORE is then extended to a localized-MISCORE, denoted by *r_l_*(*K*, *M_S_*), that can be written as,

(10) $$r_{l}\left(K,\;M_{S}\right)=\overset{k-w+1}{\underset{j=1}{\sum }}g_{j}\left(\frac{d\left(\beta_{j}\left(K\right),\beta_{j}\left(M_{S}\right)\right)}{d\left(\beta_{j}\left(K\right),\beta_{j}\left(M_{ref}\right)\right)}\right),$$

where *β_j_*(*K*), *β_j_*(*M_S_*) and *β_j_*(*M_ref_*) are the *j^th ^*local block in the *K*, the *M_S _*and the background model *M_ref_*, respectively. A *w*-length local block *β_j_*(.) can be produced by shifting a small matrix window *β*_[4×*w*] _such that (2 ≤ *w *<*k*) in the *K*, the *M_S _*and the *M_ref _*so that, *k *- *w *+ 1 number of blocks can be produced.

The weight *g_j _*for the *j^th ^*block in *M_S _*(i.e., *β_j_*(*M_S_*)) can be assigned as,

(11) $$g_{j}=\frac{G\left(\beta_{j}\left(M_{S}\right)\right)}{\sum_{q=1}^{k-w+1}G\left(\beta_{q}\left(M_{S}\right)\right)},$$

where *G*(*β_j_*(*M_S_*)) is a modified Gini purity index (a complement of the *Gini *impurity index) that can be evaluated by,

(12) $$G\left(\beta_{j}\left(M_{S}\right)\right)=\frac{1}{w}\overset{j+w-1}{\underset{i=j}{\sum }}\underset{\forall b_{i}\in \chi }{\sum }{\left(\frac{f\left(b_{i},i\right)}{p\left(b_{i}\right)}\right)}^{2},$$

where *p*(*b_i_*) is a background frequency of the base *b_i_*. Inspired by IC, *G*(*β_j_*(*M_S_*)) can characterize the conservation and the rareness properties of a block. Then, a localized-MMS with notation *R_l_*(*S*), for evaluating the merit of a set of *k*-mers *S *as a potential motif, can be given by,

(13) $$R_{l}\left(S\right)=\frac{1}{\left|S\right|}\underset{\forall K\in S}{\sum }r_{l}\left(K,\;M_{S}\right),$$

where *r_l_*(*K*, *M_S_*) is the localized-MISCORE given by Eq (10).

Note that the localized-MMS aims to improve the discrimination power for weak motifs, while it performs closely to the MMS for the strong motifs.

## Results and discussion

In this section, we evaluate the separability and the recognizability performances of MISCORE with comparison to IC and MAP score. The latter portion of the recognizability analysis describes how our MISCORE can perform motif-to-motif similarity computation and incorporate *pk *models in recognizing functional motifs.

### Separability

It is interesting to observe the performance of MISCORE, IC and MAP score in terms of separating functional motifs from the random ones. Hence, a separability performance evaluation on these modeling metrics are conducted, where the separability is considered as a metric to measure the discriminative score-gaps (normalized) between a functional motif model and a large collection of random non-functional ones.

#### Separability metric

*Sep*(*, *) score compares two metrics to learn which one has stronger discriminative power to distinguish a true motif from the random models. Given two metrics *A *and *B*, a true motif *S_t _*and a large collection of random models $\left(S_{r_{q}},\;for\;q=1,2,3,\ldots ,N\right)$, *Sep*(*A*, *B*) can be defined by

(14) $$Sep\left(A,\;B\right)=E\left\{1-\frac{\gamma_{A}\left[A\left(S_{t}\right)-A\left(S_{r_{q}}\right)\right]}{\gamma_{B}\left[B\left(S_{t}\right)-B\left(S_{r_{q}}\right)\right]}\right\},$$

where *E*{*} represents the mathematical expectation, *γ_A _*= [*A_max _*- *A_min_*]^-1^, *γ_B _*= [*B_max _*- *B_min_*]^-1^, and $\left[A\left(S_{t}\right)-A\left(S_{r_{q}}\right)\right]$ is the score-gap produced by metric *A *for *S_t _*and $S_{r_{q}}$, $\left[B\left(S_{t}\right)-B\left(S_{r_{q}}\right)\right]$ reads similarly for the metric *B*. *A_max_*(*A_min_*) and *B_max_*(*B_min_*) are the metric-specific maximum (minimum), i.e., the best (worst) possible scores, that perform a normalization. *Sep*(*A*, *B*) > 0 score interprets that the metric *B *outperforms the metric *A*, and *Sep*(*A*, *B*) < 0 score indicates the opposite case, while *Sep*(*A*, *B*) = 0 score indicates an equal separability performance by the two metrics.

For each dataset, firstly a true motif *S_t _*is generated by carefully aligning all known binding sites using CLUSTAL W [31]. Then, *N *= 5000 random models are generated by collecting random *k*-mers from the dataset and by carefully avoiding overlap with the true binding sites subject to $\left|S_{r_{q}}|\;=\;|S_{t}\right|$. The metric bounds, i.e., the best and the worst possible scores, for score normalization is required in Eq (14). The best-possible score (upper bound) of a metric can be obtained by ensuring the maximum quantification of the motif characteristics. To find the upper bound of a metric, we assume that there exist a hypothetical set of *k*-mers S^* ^that can ensure the best-possible score of a metric. With an assumption of a perfect conservation between the motif instances, i.e., $\delta \left(K_{a}^{*},\;K_{b}^{*}\right)=0$, $\forall K_{a,b}^{*}\in S^{*}$, where *δ*(*, *) is a similarity quantification, the upper bound for the metrics can be deduced using their respective equation. However, the lower bound (i.e., the worst-possible score) of the metrics are difficult to be computed since the conservation characteristic of a given motif can not be completely eliminated in any situation. Having no viable solution to compute this, the lower-bound of these metrics are practically approximated by the worst score produced by the metrics over a large collection of random models.

#### Separability results

The datasets used in this paper are split into three groups based on their origins. The first data group (denoted as *dg*_1_) contains 8 datasets that are composed of 200*bp *promoters that contain the known binding sites (functional motifs) associated with the following TFs: ERE, MEF2, SRF, CREB, E2F, MYOD, TBP and CRP. The whole datasets were collected from [32], and each dataset contains a varying number of sequences and a verified motif with known location of the binding sites. The second group (*dg*_2_) contains 20 mixed datasets (real and artificial) with 500*bp *~ 2000*bp *sequences that were collected from [10]. The third group (*dg*_3_) contains 5 datasets that are composed of 500*bp *promoters with known binding sites associated with the following TFs: CREB, SRF, TBP, MEF2 and MYOD. The 500bp promoters were collected from the Annotated regulatory Binding Sites (ABS, v1.0) database [33]. Details on these 33 datasets are presented in Table 2.

**Table 2:** Description of the used 33 datasets

*TF*	*L_seq _*(*bp*)	*Res*	*L_bs_*(*min*, *max*, *round*(*avg*))	*N_seq_*	*N_bs_*
*data group *1 (*dg*_1_): 8 real datasets [32]					

CREB	200	H	(05, 30, 12)	17	19
SRF	200	H	(09, 22, 12)	20	35
TBP	200	H	(05, 24, 07)	95	95
MEF2	200	H	(07, 15, 10)	17	17
MYOD	200	H	(06, 06, 06)	17	21
ERE	200	M	(13, 13, 13)	25	25
E2F	200	M	(11, 11, 11)	25	27
CRP	105	E	(22, 22, 22)	18	24

*data group *2 (*dg*_2_): 20 artificial datasets [10]					

dm01g	1500	D	(13, 28, 20)	04	07
dm04m	2000	D	(10, 26, 15)	04	09
hm02r	1000	H	(10, 36, 23)	09	11
hm03r	1500	H	(14, 46, 27)	10	15
hm06g	500	H	(06, 14, 08)	09	09
hm08m	500	H	(05, 34, 15)	15	13
hm09g	1500	H	(07, 26, 16)	10	10
hm10m	500	H	(07, 09, 08)	06	11
hm11g	1000	H	(06, 42, 14)	08	19
hm16g	3000	H	(09, 54, 23)	07	07
hm17g	500	H	(10, 18, 15)	11	10
hm20r	2000	H	(06, 71, 17)	35	76
hm21g	1000	H	(10, 23, 13)	05	07
hm24m	500	H	(08, 18, 12)	08	08
hm26m	1000	H	(11, 36, 25)	09	10
mus02r	1000	M	(10, 33, 19)	09	12
mus10g	1000	M	(05, 28, 15)	13	15
mus11m	500	M	(06, 27, 15)	12	15
yst08r	1000	M	(12, 49, 21)	11	14
yst09g	1000	Y	(09, 19, 17)	16	13

*data group *3 (*dg*_3_): 5 real datasets [33]					

CREB	500	H	(05, 30, 12)	17	19
SRF	500	H	(09, 22, 12)	20	36
TBP	500	H	(05, 24, 07)	95	95
MEF2	500	H	(07, 15, 10)	17	17
MYOD	500	H	(06, 06, 06)	17	21

Notations: *L_seq _*denotes the average length of the sequences in base pair count (bp), *Res *is the resource: (D, H, M, Y, E) refer to (drosophila melanogaster, (human, mouse, rat), saccharomyces cerevisiae, e.coli) respectively, *L_bs _*denotes the length of the binding sites in *bp*, *N_seq _*is the number of the sequences in the dataset and *N_bs _*is the number of the binding sites in the dataset.

First of all, *Sep*(*R*, *R_l_*) scores are computed to evaluate the improvement of the localized version. Several criteria for the local block-length (*w*) selection have been examined; and the *Sep*(*R*, *R_l_*) scores are presented in Table 3, showing that the localized version is likely to perform favorably with a smaller *w*, e.g., *w *= *round*(*k*/3), since *Sep*(*R*, *R_l_*) > 0 holds for most of the datasets. As *w *becomes larger and gets closer to *k*, the *Sep*(*R*, *R_l_*) scores tend to be zero, which makes sense in logic.

**Table 3:** *Sep*(*R*, *R_l_*) score comparison for different local block length *w *in *R_l_*

	*Sep*(*R*, *R_l_*) ± *E*{*std*} using 5000 random models			
	
TF	*w *= *O*(*k*/3)	*w *= max{*O*(*k*/3), 3}	*w *= min{*O*(*k*/2), 5}	*w *= *O*(*k*/2)
*data group *1 (*dg*_1_)				

CREB	0.022 ± 0.047	0.022 ± 0.047	-0.016 ± 0.049	-0.016 ± 0.049
SRF	-0.022 ± 0.034	-0.022 ± 0.034	-0.030 ± 0.035	-0.030 ± 0.035
TBP	0.125 ± 0.020	0.128 ± 0.020	0.128 ± 0.020	0.128 ± 0.020
MEF2	0.358 ± 0.041	0.358 ± 0.041	0.367 ± 0.041	0.367 ± 0.041
MYOD	0.066 ± 0.037	-0.089 ± 0.045	-0.089 ± 0.045	-0.089 ± 0.045
ERE	-0.008 ± 0.028	-0.008 ± 0.028	-0.081 ± 0.031	-0.210 ± 0.038
E2F	0.110 ± 0.027	0.110 ± 0.027	0.127 ± 0.026	0.136 ± 0.026
CRP	0.052 ± 0.028	0.052 ± 0.028	0.110 ± 0.024	-0.110 ± 0.039

*avg*	**0.088 **± 0.033	0.069 ± 0.034	0.065 ± 0.034	0.022 ± 0.037

*data group *2 (*dg*_2_)				

dm01g	0.101 ± 0.035	0.101 ± 0.035	0.105 ± 0.036	0.100 ± 0.037
dm04m	0.053 ± 0.033	0.053 ± 0.033	0.051 ± 0.035	0.051 ± 0.035
hm02r	0.219 ±0.043	0.219 ± 0.043	0.146 ± 0.050	0.146 ± 0.050
hm03r	0.135 ± 0.037	0.135 ± 0.037	0.146 ± 0.037	0.146 ± 0.037
hm06g	0.139 ± 0.051	0.062 ± 0.058	0.062 ± 0.058	0.062 ± 0.058
hm08m	0.084 ± 0.041	0.091 ± 0.041	0.088 ± 0.042	0.088 ± 0.042
hm09g	0.114 ± 0.075	0.114 ± 0.075	0.141 ± 0.074	0.141 ± 0.074
hm10m	0.134 ± 0.038	0.134 ± 0.038	0.129 ± 0.040	0.129 ± 0.040
hm11g	0.168 ± 0.045	0.168 ± 0.045	0.191 ± 0.044	0.191 ± 0.044
hm16g	0.140 ± 0.077	0.140 ± 0.077	0.007 ± 0.098	0.007 ± 0.098
hm17g	0.065 ± 0.045	0.065 ± 0.045	0.026 ± 0.049	0.026 ± 0.049
hm20r	0.322 ± 0.023	0.322 ± 0.023	0.299 ± 0.024	0.299 ± 0.024
hm21g	0.064 ± 0.051	0.064 ± 0.051	0.060 ± 0.054	0.060 ± 0.054
hm24m	0.107 ± 0.042	0.107 ± 0.042	0.081 ± 0.045	0.081 ± 0.045
hm26m	0.265 ± 0.044	0.265 ± 0.044	0.216 ± 0.049	0.216 ± 0.049
mus02r	0.004 ± 0.119	0.004 ± 0.119	-0.273 ± 0.198	-0.273 ± 0.198
mus10g	0.350 ± 0.056	0.354 ± 0.056	0.354 ± 0.056	0.354 ± 0.056
mus11m	0.340 ± 0.042	0.340 ± 0.042	0.329 ± 0.043	0.329 ± 0.043
yst08r	0.131 ± 0.045	0.131 ± 0.045	0.118 ± 0.047	0.107 ± 0.047
yst09g	0.353 ± 0.056	0.353 ± 0.056	0.337 ± 0.058	0.333 ± 0.059

*avg*	**0.164 **± 0.050	0.161 ± 0.050	0.131 ± 0.057	0.130 ± 0.057

*data group *3 (*dg*_3_)				

CREB	0.072 ± 0.042	0.072 ± 0.042	0.049 ± 0.043	0.049 ± 0.043
SRF	-0.026 ± 0.028	-0.026 ± 0.028	-0.032 ± 0.029	-0.032 ± 0.029
TBP	0.129 ± 0.019	0.133 ± 0.019	0.133 ± 0.019	0.133 ± 0.019
MEF2	0.372 ± 0.042	0.372 ± 0.042	0.380 ± 0.042	0.380 ± 0.042
MYOD	0.088 ± 0.034	-0.076 ± 0.042	-0.076 ± 0.042	-0.076 ± 0.042

*avg*	**0.127 **± 0.033	0.095 ± 0.035	0.091 ± 0.035	0.091 ± 0.035

**Result summary:**	*E*{*Sep*(*R*, *R_l_*)} ± *E*{*std*} on each data group			

*dg*_1_	0.088 ±0.033	0.069 ± 0.034	0.065 ± 0.034	0.022 ± 0.037
*dg*_2_	0.164 ±0.050	0.161 ± 0.050	0.131 ± 0.057	0.130 ± 0.057
*dg*_3_	0.127 ±0.033	0.095 ± 0.035	0.091 ± 0.035	0.091 ± 0.035

*avg*	**0.126 **±0.039	0.108 ± 0.040	0.095 ± 0.042	0.081 ± 0.043

Remark: *O*(*) is a rounding operator and *k *is the length of *k*-mers. *Sep*(*R*, *R_l_*) is computed on each dataset using 5000 random set of *k*-mers generated from each dataset. The result summary shows that *w *= *O*(*k*/3) criterion is likely to produce a better separability performance; hence it can be generally applied in the localization approach.

A separability comparison among *R*, *R_l_*, IC and MAP score is then conducted on the 33 datasets. The results are presented in Table 4, showing that MISCORE can achieve a comparable separability performance to IC and a remarkably improved performance than MAP score, which is indicated by the average *Sep*(*, *) scores on the three data groups, that is, [*Sep*(*IC*, *R*), *Sep*(*IC*, *R_l_*), *Sep*(*MAP*, *R*), *Sep*(*MAP*, *R_l_*)]= [-0.144, 0.016, 0.273, 0.374]. In our experiments, MAP score is computed using a 3rd-order Markov chain model. A higher order Markov chain model may improve the separability performance for MAP score, however, the computational cost would be much higher in such a case.

**Table 4:** *Sep*(*, *) score comparison among *R*, *R_l_*, *IC *and *MAP *score

Result details: *Sep*(*, *) ± *E*{*std*} on each dataset using 5000 random models						
***dg***	***TF***	***Sep*(*IC*, *R*)**	***Sep*(*IC*, *R_l_*)**	***Sep*(*MAP*, *R*)**	***Sep*(*MAP*, *R_l_*)**	***Sep*(*R*, *R_l_*)**

	CREB	-0.099 ± 0.051	-0.080 ± 0.013	0.255 ± 0.030	0.268 ± 0.014	0.022 ± 0.047
	SRF	-0.104 ± 0.036	-0.133 ± 0.008	0.313 ± 0.020	0.294 ± 0.009	-0.022 ± 0.034
	TBP	-0.088 ± 0.025	0.056 ± 0.002	0.302 ± 0.014	0.395 ± 0.005	0.125 ± 0.020
	MEF2	-0.405 ± 0.088	0.092 ± 0.020	0.144 ± 0.049	0.446 ± 0.017	0.358 ± 0.041
*dg*_1_	MYOD	-0.113 ± 0.043	-0.022 ± 0.010	0.299 ± 0.025	0.356 ± 0.011	0.066 ± 0.037
	ERE	0.060 ± 0.027	0.057 ± 0.011	0.416 ± 0.017	0.414 ± 0.012	-0.008 ± 0.028
	E2F	-0.048 ± 0.032	0.064 ± 0.012	0.350 ± 0.018	0.419 ± 0.012	0.110 ± 0.027
	CRP	0.013 ± 0.032	0.070 ± 0.018	0.486 ± 0.018	0.516 ± 0.013	0.052 ± 0.028

	*avg*	-0.098 ± 0.042	**0.013 **± 0.012	**0.321 **± 0.024	**0.388 **± 0.012	**0.088 **± 0.033

	dm01g	-0.080 ± 0.042	0.024 ± 0.027	0.294 ± 0.024	0.361 ± 0.023	0.101 ± 0.035
	dm04m	-0.029 ± 0.038	0.026 ± 0.025	0.350 ± 0.022	0.384 ± 0.022	0.053 ± 0.033
	hm02r	-0.187 ± 0.067	0.089 ± 0.029	0.320 ± 0.037	0.478 ± 0.024	0.219 ± 0.043
	hm03r	-0.096 ± 0.045	0.076 ± 0.017	0.276 ± 0.026	0.389 ± 0.015	0.135 ± 0.037
	hm06g	-0.145 ± 0.068	0.001 ± 0.031	0.227 ± 0.040	0.325 ± 0.025	0.139 ± 0.051
	hm08m	-0.006 ± 0.048	0.082 ± 0.024	0.277 ± 0.030	0.340 ± 0.021	0.084 ± 0.041
	hm09g	-0.120 ± 0.087	-0.009 ± 0.041	0.211 ± 0.053	0.288 ± 0.035	0.114 ± 0.075
	hm10m	-0.070 ± 0.050	0.071 ± 0.027	0.290 ± 0.030	0.383 ± 0.022	0.134 ± 0.038
*dg*_2_	hm11g	-0.172 ± 0.062	0.077 ± 0.016	0.224 ± 0.036	0.388 ± 0.016	0.168 ± 0.045
	hm16g	-0.218 ± 0.100	0.000 ± 0.049	0.227 ± 0.056	0.364 ± 0.038	0.140 ± 0.077
	hm17g	-0.076 ± 0.052	-0.022 ± 0.026	0.379 ± 0.029	0.409 ± 0.021	0.065 ± 0.045
	hm20r	-0.344 ± 0.044	0.098 ± 0.002	0.234 ± 0.022	0.486 ± 0.006	0.322 ± 0.023
	hm21g	-0.183 ± 0.062	-0.075 ± 0.036	0.293 ± 0.035	0.357 ± 0.027	0.064 ± 0.051
	hm24m	-0.082 ± 0.052	0.024 ± 0.032	0.324 ± 0.031	0.390 ± 0.026	0.107 ± 0.042
	hm26m	-0.114 ± 0.067	0.177 ± 0.034	0.377 ± 0.039	0.540 ± 0.028	0.265 ± 0.044
	mus02r	-0.034 ± 0.110	-0.061 ± 0.058	0.409 ± 0.062	0.393 ± 0.046	0.004 ± 0.119
	mus10g	-0.630 ± 0.134	-0.052 ± 0.020	0.001 ± 0.076	0.355 ± 0.019	0.350 ± 0.056
	mus11m	-0.623 ± 0.098	-0.049 ± 0.021	0.050 ± 0.054	0.386 ± 0.019	0.340 ± 0.042
	yst08r	-0.019 ± 0.050	0.149 ± 0.024	0.037 ± 0.040	0.196 ± 0.019	0.131 ± 0.045
	yst09g	-0.253 ± 0.102	0.179 ± 0.036	-0.053 ± 0.073	0.310 ± 0.029	0.353 ± 0.056

	*avg*	-0.174 ± 0.069	**0.040 **± 0.029	**0.237 **± 0.041	**0.376 **± 0.024	**0.164 **± 0.050

	CREB	-0.102 ± 0.047	-0.056 ± 0.012	0.248 ± 0.028	0.280 ± 0.013	0.072 ± 0.042
	SRF	-0.085 ± 0.029	-0.131 ± 0.007	0.324 ± 0.016	0.296 ± 0.008	-0.026 ± 0.028
*dg*_3_	TBP	-0.080 ± 0.023	0.052 ± 0.002	0.307 ± 0.013	0.392 ± 0.005	0.129 ± 0.019
	MEF2	-0.420 ± 0.092	0.122 ± 0.020	0.132 ± 0.051	0.463 ± 0.017	0.372 ± 0.042
	MYOD	-0.115 ± 0.040	-0.017 ± 0.009	0.297 ± 0.023	0.358 ± 0.010	0.088 ± 0.034

	*avg*	-0.160 ± 0.046	-0.006 ± 0.010	**0.262 **± 0.026	**0.358 **± 0.011	**0.127 **± 0.033

**Result summary**: *E*{*Sep*(*, *)} **± ***E*{*std*} on each data group						

	data group (*dg*)	*Sep*(*IC*, *R*)	*Sep*(*IC*, *R_l_*)	*Sep*(*MAP*, *R*)	*Sep*(*MAP*, *R_l_*)	*Sep*(*R*, *R_l_*)

	*dg*_1_	-0.098 ± 0.042	0.013 ± 0.012	0.321 ± 0.024	0.388 ± 0.012	0.088 ± 0.033
	*dg*_2_	-0.174 ± 0.069	0.040 ± 0.029	0.237 ± 0.041	0.376 ± 0.024	0.164 ± 0.050
	*dg*_3_	-0.160 ± 0.046	-0.006 ± 0.010	0.262 ± 0.026	0.358 ± 0.011	0.127 ± 0.033

	*avg*	-0.144 ± 0.052	**0.016 **± 0.017	**0.273 **± 0.030	**0.374 **± 0.015	**0.126 **± 0.039

Remark: *Sep*(*, *) score is computed on a dataset using 5000 random set of *k*-mers generated from the dataset. It can be seen that the localized version improves MISCORE in terms of separability performance, i.e., *Sep*(*R*, *R_l_*) > 0 holds for most of the cases. *Sep*(*, *) score comparison among other metrics show that MISCORE is likely to produce favorable separability performance than IC and MAP score.

### Recognizability

It is often observed that after evaluating a set of candidate motifs returned by a discovery tool, the top ranked candidates are not necessarily functional. The ineffectiveness of the motif evaluation metric used can be one of the reasons behind this. Therefore, we have conducted a recognizability performance comparison among these metrics.

Recognizability refers to how well a metric can recognize the best candidate motif from a set of putative candidates in terms of ranking, where the best candidate motif is expected to be top ranked. To conduct this evaluation, we need to have a set of putative candidate motifs generated by some motif discovery tools on each dataset. In this study, we employed MEME [34] to generate a set of putative motifs for each dataset. Then, the best candidate motif is identified by the *F*-measure [35]: *F *= 2*PR*/(*P *+ *R*), where *P *= *TP*/(*TP *+ *FP*), *R *= *TP*/(*TP *+ *FN*), where *TP*, *FP *and *FN *are the number of true positive, false positive and the false negative predictions, respectively. *TP *refers to the number of the true binding sites overlapped by at least one predicted site. In this study, we considered a true positive count if a true binding site is overlapped by a predicted site with at least 25% of the length of the true site. *FP *is the number of the predicted sites that do not have more than 25% overlap with any true binding sites; and *FN *is the number of the true binding sites that are not overlapped by any predicted sites with at least 25% of the length of the binding site.

These candidate motifs for each dataset are then scored by IC, MAP score, *R*, and *R_l _*respectively, and ranked according to their scores. The assigned rank of the best motif is recorded for each dataset in order to find that which metric can assign a comparatively higher rank to the best motif. In order to evaluate the ranking order, the following criterion is adopted to compute a *mean rank *(*μ*) score [36]:

(15) $$\mu =\frac{Q\left(Q+1\right)}{2\sum_{i=1}^{Q}rank\;\left(M_{i}\right)},$$

where *Q *is the number of the relevant items whose rank orders are to be considered. In our case, only the best motif's rank is considered, hence *Q *= 1 and Eq (15) becomes *μ *= 1/*rank*(*best motif*).

An average *μ *score over 10 runs with each metric on each dataset is recorded using a set of candidate motifs produced by MEME during each run. The results are presented in Table 5, which also includes a data group-wise *E*{*μ*} score as result summary showing that both *R *and *R_l _*offer a considerably better recognizability than MAP score, while IC is likely to perform the best recognizability performance. However, we observed that a 10-run average *μ *score computed using *dg*_1 _and *dg*_2 _(i.e., 28/33 datasets) indicates that both *R *and *R_l _*can outperform IC and MAP score.

**Table 5:** Recognizability scores for the *best *candidate motifs

Result details: a 10-run average *μ *score on each dataset					
**data group (*dg*)**	***TF***	***MAP***	***IC***	***R***	***R_l_***

	CREB	0.339	0.433	0.383	0.384
	SRF	0.582	0.757	0.725	0.721
	TBP	0.529	0.717	0.750	0.800
	MEF2	0.362	0.763	0.742	0.757
*dg*_1_	MYOD	0.517	0.265	0.243	0.209
	ERE	0.512	0.750	0.875	1.000
	E2F	0.383	0.800	0.800	0.700
	CRP	1.000	1.000	1.000	1.000

*avg*		0.528	0.686	**0.690**	**0.696**

	dm01g	0.107	0.195	0.151	0.127
	dm04m	0.180	0.134	0.219	0.188
	hm02r	0.159	0.305	0.700	0.617
	hm03r	0.257	0.179	0.225	0.255
	hm06g	0.264	0.176	0.255	0.297
	hm08m	0.341	0.304	0.224	0.320
	hm09g	0.156	0.299	0.304	0.307
	hm10m	0.364	0.416	0.489	0.474
*dg*_2_	hm11g	0.275	0.390	0.194	0.192
	hm16g	0.419	0.540	0.550	0.507
	hm17g	1.000	1.000	1.000	1.000
	hm20r	0.456	0.304	0.306	0.390
	hm21g	0.407	0.450	0.180	0.190
	hm24m	0.198	0.172	0.263	0.266
	hm26m	0.297	0.313	0.317	0.169
	mus02r	0.400	0.393	0.233	0.332
	mus10g	1.000	0.867	0.900	0.800
	mus11m	0.254	0.392	0.532	0.558
	yst08r	0.247	0.239	0.151	0.231
	yst09g	0.389	0.460	0.344	0.314

*avg*		0.359	0.376	**0.377**	**0.377**

	CREB	0.512	0.422	0.375	0.540
	SRF	0.369	0.407	0.373	0.398
*dg*_3_	TBP	0.542	0.875	0.583	0.750
	MEF2	0.533	1.000	0.467	0.433
	MYOD	0.488	0.425	0.453	0.400

*avg*		0.489	0.**626**	0.450	0.504

**Result summary: **a 10-run average *μ *score on each data group					

*dg*_1_		0.528	0.686	0.690	0.696
*dg*_2_		0.358	0.376	0.377	0.377
*dg*_3_		0.489	0.626	0.450	0.504

*avg*{*dg*_1_, *dg*_2_, *dg*_3_}		0.458	**0.563**	0.506	0.526
*avg*{*dg*_1_, *dg*_2_}		0.443	0.531	0.533	**0.536**

Remark: a higher *μ *score indicates a better ability of a metric in recognizing the best candidate motif in terms of rank order from a set of putative motifs returned by a tool. MISCORE is found to have convincing recognizability performances that are comparable to IC and remarkably better than MAP score as indicated in the result summary.

#### Recognizability on degenerated motifs

Weak motif characterization and recognition is challenging to all evaluation metrics. Therefore, in order to observe how the considered metrics perform in recognizing degenerated motifs, we first split the 33 datasets into two categories, i.e., strong and weak motif classes, based on the average positional conservation of the motif PFMs, which is defined as $apc\left(S_{t}\right)=\frac{1}{k}\sum_{i=1}^{k}\underset{b_{i}}{\text{max}}\left\{f\left(b_{i},\;i\right)\right\}$, *b_i _*∈ {*A*, *C*, *G*, *T*}.

Table 6 reports the average recognizability scores of these metrics on the datasets. The results show that MISCORE can noticeably outperform MAP score and perform comparably to IC in recognizing weak motifs. However, IC outperforms our MISCORE and MAP score in recognizing strong motifs.

**Table 6:** Strong/weak motif class-wise average recognizability scores

Strong/weak motif class-wise *E *{*μ*}over 10 runs					
**Motif class**	***apc*(*S_t_*) *range***	***MAP***	***IC***	***R***	***R_l_***

**Weak **(17/33 datasets)	*apc *≤0.75	0.373	0.412	0.409	**0.436**
Strong (16/33 datasets)	*apc *>0.75	0.463	**0.562**	0.516	0.507

Remark: recognizability scores obtained by the metrics are compared between strong and weak motifs. Results show that MISCORE noticeably outperforms MAP score and performs comparably to IC in recognizing weak motifs. However, the localized-MISCORE is likely to be more effective in recognizing weak motifs than IC and MAP score.

#### Motif recognition using priori-known models

If there exists priori known (*pk*) estimation of the target motif profile during the search in the query sequences, then the motif discovery algorithms can greatly benefit by utilizing such *a priori *knowledge in finding motifs that have similar characteristics to the *pk *model. Often a priori estimation of a target motif model can be obtained from the public databases e.g., [37-39], or by collecting a set of binding sites from the sequences that are known to be co-regulated by the target TF [29]. These *pk *models can only be the estimation of the target motifs in the search, since: (i) the known binding sites in the public databases are usually incomplete, which may cause the *pk *profiles to have an incomplete representation that may not be able to reliably discriminate a true motif from a false one [40], and (ii) due to the sequence dissimilarity between the query sequences and the sequences that are known to be co-regulated by the target TF.

One plausible use of the *pk *models is their involvement in the process of motif evaluation, where the putative motifs will be recognized by referring to the *pk *models. The ranking of the candidate motifs then becomes a motif-to-motif similarity quantification between the putative and the *pk *models.

MAP score is unable to evaluate the motif-to-motif similarity. IC, on the other hand, is not originally meant for motif-to-motif similarity computation. However, it has been extended as the average log likelihood ratio (ALLR) [41] for this task. Several other metrics can perform motif-to-motif similarity quantification, e.g., Pearson correlation coefficient (PCC) [42], Kullback-Leibler divergence (KLD) [43-45], Euclidean distance (ED) [46] and Sandelin-Wasserman (SW) metric [47]. But, these metrics can only compute a motif-to-motif similarity without considering motif characteristics.

Motivated by the above facts, MISCORE framework is examined to perform the motif-to-motif similarity while taking account of the motif characterization. Let a candidate motif *S *be ranked by using a *pk *model *M_pk_*. Then, MISCORE becomes

(16) $$r_{pk}\left(K,\;M_{pk}\right)=\frac{d\left(K,M_{pk}\right)}{d\left(K,M_{ref}\right)+c\left(K\right)}.$$

The MMS score (*R*) given in Eq (9) then can be written as,

(17) $$R_{pk}\left(S\right)=\frac{1}{\left|S\right|}\underset{\forall K\in S}{\sum }r_{pk}\left(K,\;M_{pk}\right).$$

Note that *R_pk _*and *r_pk_*, characterizing motif signals with assistance of *pk *models, can be regarded as the supervised counterparts of *R *and *r*, respectively. localized-MISOCRE can be expressed to accommodate the *pk *models in a similar manner. Similarly, MISCORE can be employed to compute the motif-to-motif similarity in order to group similar candidate motifs in the relevant applications.

For simplicity, we demonstrate that MISCORE with the use of *pk *models can help in recognizing putative motifs, and performs favorably against other metrics. To do this, we first generated a *pk *model for each dataset by extracting the non-redundant known binding sites associated with CREB, E2F, MEF2 and SRF transcription factors from JASPAR [37]; ERE, MYOD and TBP from TRANSFAC (public v7.0) [38]; and CRP from RegulonDB [39] databases. After alignment, the *pk *models are generated for the datasets in *dg*_1 _and *dg*_3 _since they share common transcription factors. For the 20 datasets in *dg*_2_, we applied a multiple sequence alignment tool GLAM [48] to align the binding sites of each dataset. Then, the longest conserved block from the alignment is extracted to form a *pk *model for each dataset.

The data group-wise average recognizability scores obtained by the metrics over 10 runs are presented in Table 7, showing that MISCORE others a promising performance with comparison to other metrics in terms of recognizing the best candidate motifs using the *pk *models.

**Table 7:** Recognizability scores for the best candidate motifs using *pk *models

Result details: a 10-run average *μ *score on each dataset								
**data group (*dg*)**	**TF**	***R_pk_***	**$R_{l_{pk}}$**	**PCC**	**ALLR**	**KLD**	**ED**	**SW**

	CREB	0.339	0.333	0.096	0.295	0.275	0.370	0.080
	SRF	0.667	0.717	0.500	0.553	0.553	0.657	0.564
	TBP	1.000	1.000	1.000	1.000	1.000	1.000	1.000
	MEF2	1.000	1.000	1.000	1.000	1.000	1.000	1.000
*dg*_1_	MYOD	0.645	0.651	0.665	0.656	0.656	0.656	0.640
	ERE	1.000	1.000	1.000	1.000	0.917	0.875	1.000
	E2F	1.000	1.000	1.000	1.000	1.000	1.000	1.000
	CRP	1.000	1.000	1.000	1.000	1.000	1.000	0.792

	*avg*	0.831	0.837	0.783	0.813	0.800	0.820	0.760

	dm01g	0.667	0.667	0.342	0.528	0.694	0.722	0.371
	dm04m	0.377	0.485	0.662	0.498	0.487	0.484	0.647
	hm02r	0.800	0.700	1.000	0.547	0.447	0.447	1.000
	hm03r	0.255	0.425	0.690	0.514	0.514	0.300	0.556
	hm06g	0.444	0.429	0.611	0.407	0.353	0.546	0.427
	hm08m	0.861	0.861	0.852	0.854	0.771	0.857	0.857
	hm09g	0.539	0.565	0.205	0.389	0.512	0.556	0.285
	hm10m	0.412	0.495	0.558	0.490	0.490	0.500	0.820
*dg*_2_	hm11g	0.302	0.329	0.829	0.335	0.285	0.333	0.829
	hm16g	0.690	0.767	0.105	0.617	0.767	0.900	0.100
	hm17g	1.000	1.000	1.000	1.000	1.000	1.000	1.000
	hm20r	0.537	0.537	0.708	0.542	0.542	0.548	0.708
	hm21g	0.148	0.148	0.483	0.204	0.214	0.214	0.324
	hm24m	0.573	0.650	1.000	0.592	0.592	0.725	0.867
	hm26m	0.450	0.650	0.369	0.650	0.567	0.617	0.700
	mus02r	0.182	0.209	0.329	0.184	0.184	0.199	0.345
	mus10g	1.000	1.000	1.000	1.000	1.000	1.000	1.000
	mus11m	1.000	1.000	1.000	1.000	1.000	1.000	1.000
	yst08r	0.567	0.633	0.524	0.567	0.583	0.580	0.767
	yst09g	0.201	0.232	0.292	0.179	0.186	0.217	0.321

	*avg*	0.550	0.589	0.628	0.555	0.559	0.587	0.646

	CREB	0.642	0.642	0.556	0.657	0.657	0.667	0.476
	SRF	0.667	0.667	0.523	0.707	0.650	0.667	0.822
*dg*_3_	TBP	1.000	1.000	1.000	1.000	1.000	1.000	1.000
	MEF2	0.653	0.656	0.656	0.750	0.850	0.662	0.482
	MYOD	0.486	0.653	0.500	0.563	0.563	0.577	0.661

	*avg*	0.690	0.723	0.647	0.735	0.744	0.715	0.688

**Result summary: **a 10-run average *μ *score on each data group								

*dg*_1_		0.831	0.837	0.783	0.813	0.800	0.820	0.760
*dg*_2_		0.550	0.589	0.628	0.555	0.559	0.587	0.646
*dg*_3_		0.690	0.723	0.647	0.735	0.744	0.715	0.688

*avg*		0.690	**0.717**	0.686	0.701	0.701	0.707	0.698

Remark: MISCORE metrics *R_pk _*and $R_{l_{pk}}$compute motif-to-*pk *similarity through the characterization of the motif signals, while the other metrics can not perform motif characterization. The result summary shows that MISCORE is capable of effectively utilizing the *pk *models in recognizing the functional motifs. Note: PCC: Pearson correlation coefficient [42]; ALLR: average log likelihood ratio [41]; KLD: Kullback-Leibler divergence [43-45]; ED: Euclidean distance [46]; and SW: Sandeline-Wasserman metric [47].

### Background rareness and over-representation

Another key concept in computational motif discovery is over-representation [13-15,49]. It looks for motifs that have significant occurrences in the query sequences (input promoters) than the background sequences through some statistical quantification [13,16]. The functionality of this site multiplicity, i.e., 'the shadow appearances of the binding sites', in the regulatory regions could constitute a mechanism for lateral diffusion of the transcription factors along the sequences, and/or the shadow sites might be the fossils from the process of binding site turnover [16,50]. Even though the biological reasons behind this site multiplicity are yet to be fully understood [16], it is often considered as a useful motif characteristic and well recognized in the working field.

It is interesting to analyze the correlation between a functional motif's background rareness and over-representation, although both can partially characterize the functional motifs. This section tries to make a sensible link between these two key concepts.

### Correlation between background rareness and over-representation

Our aim is to show how MISCORE can be used to characterize a motif's background rareness through its over-representation feature using foreground (i.e., promoters) and background information. We first define a constrained frequency (*cf*) measure in order to compute an occurrence score of a given motif using MISCORE. Given a set *S_all _*to contain all possible *k*-mers from a set of sequences (either foreground or background) and a motif *S *with a PFM model *M_S_*, *cf *is defined as:

(18) $$cf\left(M_{S},\;S_{all}\right)=\frac{\left|T\right|}{\left|S_{all}\right|},where\;T=\left\{\forall K\in S_{all}\;:\;r\left(K,\;M_{S}\right)\leq \theta \right\},$$

where | * | represents the set cardinality, *r*(*K*, *M_S_*) is the MISCORE given in Eq (6) and *θ *is a cut-off threshold that can be defined as *θ *= *R*(*S*) + *std*(*S*)λ, where *std *represents the standard deviation operator, λ is a threshold regulator and *R*(*) is the MMS given in Eq (9).

Regulatory regions often contain more frequent occurrences of a functional motif compare to the sequence-backgrounds, due to the mutational constraints in the foreground compared to the backgrounds. Hence, a true motif is expected to produce a larger *cf *in the promoter regions (foreground) than the backgrounds for a given similarity threshold. Therefore, the MISCORE-based over-representation score *ORS_r _*for a motif *S *can be given using Eq (18) as,

(19) $$ORS_{r}\left(M_{S}\right)=\frac{cf\left(M_{S},S_{bg}\right)}{cf\left(M_{S},S_{fg}\right)},$$

where *S_bg _*and *S_fg _*are the sets of all *k*-mers produced by window shifting in the background and in the foreground regions, respectively.

The condition *ORS_r_*(*M_S_*) < 1 indicates that *M_S _*has a higher frequency in the foreground than the background for a given threshold, which implies that there are comparatively less occurrences of that motif in the background (i.e., background rareness) than the foreground. Hence, the background rareness of a motif can be characterized through its over-representation feature, that can be statistically quantified.

## Demonstration

We collected the background sequences for CREB, SRF, TBP, MEF2 and MYOD datasets from public databases (e.g., http://www.ncbi.nlm.nih.gov and http://www.ebi.ac.uk) as the respective sequence backgrounds. The respective 200*bp *and 500*bp *promoter regions are then taken as the sequence-foregrounds for each TF. The *ORS_r_*(*M_t_*) scores for different thresholds are computed for each TF and presented in Table 8, showing that the background rareness can be characterized through the over-representation of the functional motifs since *ORS_r_*(*M_t_*) < 1 holds for all cases. It also shows that, as the promoter region grows in length from 200*bp *to 500*bp*, the *ORS_r _*scores tend to increase for the functional motifs, as anticipated.

**Table 8:** *ORS_r_*(*M_t_*) scores with several threshold regulators

		*ORS_r_*(*M_t_*), *θ *= *R*(*S_t_*) + *std*(*S_t_*) λ			
TF	*L_fg_*(*bp*)	**λ = -0.25**,	**λ = 0.0**,	**λ = 0.25**,	λ = 0.5
CREB	200	0.391	0.357	0.429	0.537
	500	0.762	0.576	0.884	0.806

SRF	200	0.040	0.048	0.055	0.059
	500	0.107	0.108	0.126	0.144

TBP	200	0.334	0.385	0.441	0.548
	500	0.671	0.778	0.793	0.803

MEF2	200	0.041	0.050	0.065	0.100
	500	0.129	0.177	0.392	0.655

MYOD	200	0.292	0.289	0.289	0.289
	500	0.303	0.620	0.710	0.746

Remark: MISCORE-based over-representation scores *ORS_r_*(.) are computed for each dataset with different thresholds. *ORS_r_*(*M_t_*) < 1 holds for all cases, indicating that the background rareness and the over-representation of functional motifs are correlated by MISCORE. As the promoter region grows in length from 200*bp *to 500*bp*, the *ORS_r_*(*M_t_*) scores tend to increase as anticipated. Note: *L_fg _*denotes the length of the promoter sequences.

In order to conduct a statistical evaluation, the *ORS_r_*(*M_t_*) score of the true motif of each dataset is evaluated using two large sets of (i) conserved (*M_c_*) and (ii) random models (*M_r_*). Each random model *M_r _*and conserved model *M_c _*is generated according to the criteria that have been described earlier. It has been observed that the following holds for all cases with a given similarity threshold, that is,

(20) $$ORS_{r}\left(M_{t}\right)<E\left\{ORS_{r}\left(M_{c}\right)\right\}<E\left\{ORS_{r}\left(M_{r}\right)\right\}.$$

**Figure 1:**
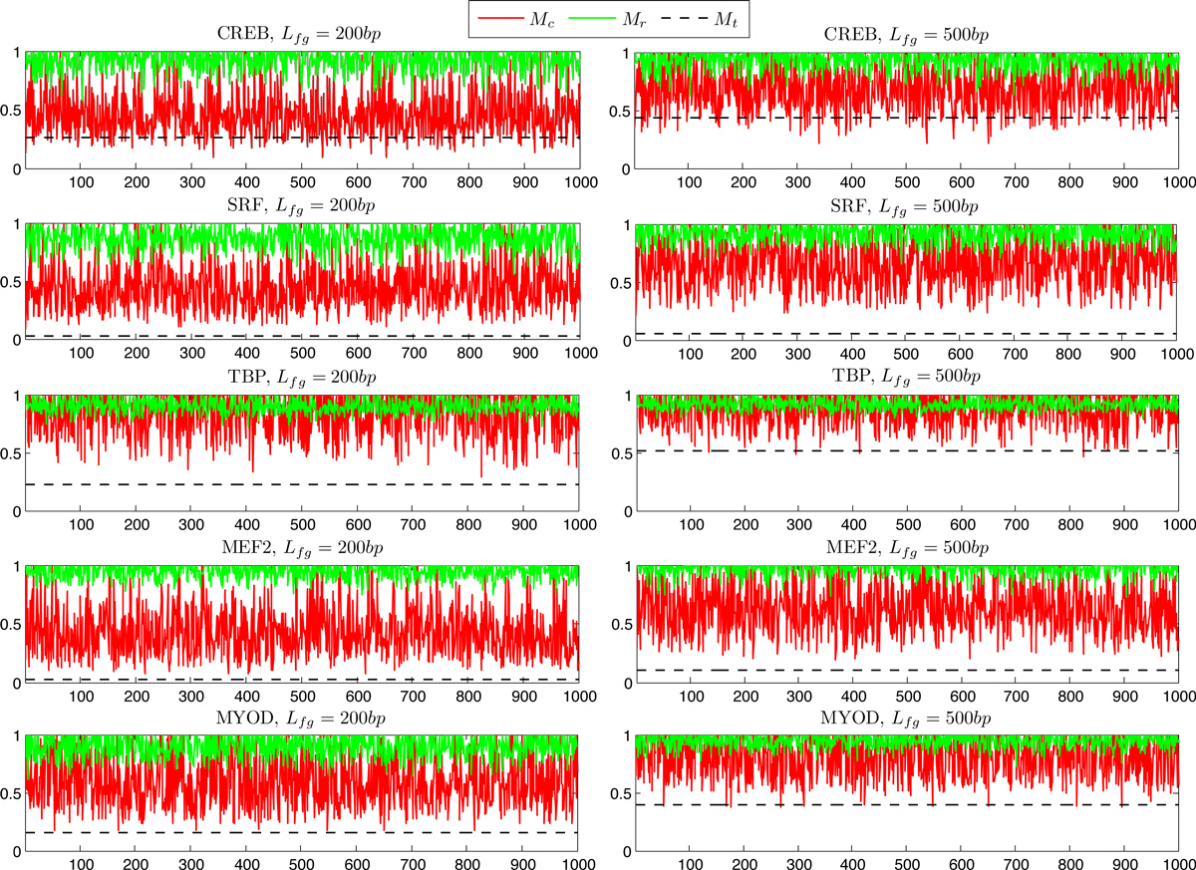
**Correlation between the over-representation and the background rareness**. *ORS_r _*scores for the functional models *M_t_*, the random models $M_{r_{q}}$, and the conserved models $M_{c_{q}}$ for *q *= 1, 2, 3, . . ., 1000 are plotted for each dataset with 200*bp *and 500*bp *promoters in the left and in the right column, respectively. Threshold *θ *= *R*(*M_t_*) + *std*(*M_t_*)λ, λ = 0.0 is used. The figure depicts a rareness interpretable visualization through the statistical over-representation property of the functional motifs by showing that, the *ORS_r_*(*M_r_*) scores are found distant from the *ORS_r_*(*M_t_*) scores for all cases which implies that the random models have close to zero chance of being over-represented with comparison to the true models. In addition to this, the *ORS_r_*(*M_t_*) scores are found to be mostly rare with comparison to the *ORS_r_*(*M_c_*) scores, i.e., these non-functional conserved models have a rare chance of having better over-representation scores than the true models, for most of the datasets.

This implies that *ORS_r_*(*M_t_*) scores are relatively rare in respect to *E*{*ORS_r_*(*M_c_*)} with a given similarity threshold. Since the *M_c _*models, despite being conserved, have less chance of being over-represented than a true model *M_t_*. In addition to this, *ORS_r_*(*M_t_*) scores are found to be the rarest with comparison to the random models *M_r_*. In other words, *E*{*ORS_r_*(*M_r_*)} >*ORS_r_*(*M_t_*) implies that, the random models have a comparatively larger background-to-foreground occurrence ratio (see Eq (19)) than the functional motifs. This characterizes the background rareness property of a functional motif through its over-representation property. Figure 1 demonstrates the correlation between the background rareness and the over-representation for 10 datasets.

## Conclusions

This paper contributes a mismatch-based fast computational tool for modeling DNA regulatory motifs. It is free from any assumption on the model dependency, and it escapes from the use of background modeling using Markov chain models. Simultaneously, it embeds the compositional complexity in modeling the motif signals. Our proposed MISCORE can be used as a metric to measure the similarity between *k*-mers and a motif model, also it can be employed to compute the motif-to-motif similarity.

The experimental results on 33 datasets indicate that MISCORE performs favorably with comparison to the well-known IC and MAP score in terms of the separability and the recognizability. These results also show that MISOCRE is functionally effective in recognizing degenerated motifs, and it can embed the *pk *models to perform candidate motif ranking.

MISCORE has good potential to be employed as a similarity metric in rule-based or clustering-based motif discovery algorithms, it can also be employed as a numerical feature in machine learning approaches for finding motifs. Furthermore, MISCORE-based Motif Score (MMS) can be employed as a fitness function in evolutionary computation approaches for motif discovery, and for candidate motif ranking in computational motif discovery tools.

## Competing interests

We declare that the authors have no competing interests.

## Authors' contributions

DW proposed and developed the MISCORE framework with original ideas and the mathematical formulas. He also directed the experimental design and performance analysis. ST mainly contributed to the development of the localized version of MISCORE and the implementation of experiments. Both authors contributed to the writing of the paper, and read and approved the final manuscript.
